# In Vitro and In Silico Evaluation of Polymyxin B Aerosol Delivery in Adult Mechanical Ventilation

**DOI:** 10.3390/pharmaceutics18010058

**Published:** 2025-12-31

**Authors:** Shengnan Zhang, Guanlin Wang, Jingjing Liu, Xuejuan Zhang, Qi Pei

**Affiliations:** 1 Department of Pharmacy, The Third Xiangya Hospital of Central South University, Changsha 410013, China; secnanzhang@gmail.com; 2 School of Pharmaceutical Sciences, Sun Yat-Sen University, Guangzhou 510006, China; wangglin3@mail2.sysu.edu.cn; 3 Department of Intensive Medicine, The Third Xiangya Hospital of Central South University, Changsha 410013, China; ivljingjing@126.com; 4 College of Pharmacy, Jinan University, Guangzhou 510006, China; 5 Xiangya School of Pharmacy, Central South University, Changsha 410013, China

**Keywords:** polymyxin B, nebulization, mechanical ventilation, pulmonary delivery, MPPD model

## Abstract

**Background:** Nebulized polymyxin B (PMB) therapy is widely used in intensive care units for treating hospital-acquired and ventilator-associated pneumonia caused by multidrug-resistant Gram-negative bacteria, yet its pulmonary delivery performance during invasive mechanical ventilation remains poorly characterized. **Methods:** An in vitro adult mechanical ventilation model was used. We evaluated two nebulizers (vibrating mesh nebulizer [VMN] and jet nebulizer [JN]) at three positions (standalone nebulizer, 15 cm from the Y-piece, and the humidifier’s dry end) with two artificial airway types (endotracheal and tracheostomy tubes). Lung deposition was predicted using the multiple-path particle dosimetry model, incorporating the Yeh/Schum five-lobe adult lung model. **Results:** In the standalone setup, the percentage of delivered dose of VMN and JN was approximately 40% and 34%, respectively. Mechanical ventilation significantly reduced the delivered dose (all *p* ≤ 0.0085), with VMN at the humidifier’s dry end delivering only 2.14–2.99% of the nominal dose. In all the tested ventilation scenarios, both the use of the JN and positioning the nebulizer 15 cm from the Y-piece significantly increased aerosol delivery (all *p* ≤ 0.021). While the ventilator circuit reduced the total drug amount, it filtered larger aerosols. This resulted in a smaller mass median aerodynamic diameter and a higher fine particle fraction (all *p* < 0.0001), which doubled the predicted alveolar deposition fraction (from 13–14% in standalone to 23–28% in ventilation scenarios) and eliminated extrathoracic deposition. **Conclusions:** This study provides the first in vitro and in silico assessment of PMB aerosol delivery during invasive mechanical ventilation. Nebulizer type, its placement within the circuit, and the artificial airway are critical factors that significantly alter the pulmonary delivery of PMB aerosol and subsequently impact its lung deposition.

## 1. Introduction

The global spread of multidrug-resistant Gram-negative bacteria (MDR-GNB) has significantly exacerbated the treatment difficulty of hospital-acquired pneumonia and ventilator-associated pneumonia [1,2,3]. Carbapenem-resistant Enterobacteriaceae, Acinetobacter baumannii, and Pseudomonas aeruginosa, classified as “Critical Priority” and “High Priority” pathogens by the World Health Organization in 2024, are particularly concerning, with associated mortality rates that can exceed 40% [4,5,6]. Although several novel antibiotics have been approved in recent years for the treatment of hospital-acquired pneumonia caused by MDR-GNB, their widespread clinical use and evaluation remain limited, and polymyxin B (PMB) continues to be one of the last-line agents against MDR-GNB infection [1,7]. However, therapeutic use is limited by two interrelated obstacles: (i) limited penetration from plasma into the epithelial lining fluid (ELF) and infected parenchyma, and (ii) a narrow therapeutic window owing to dose-dependent nephro- and neurotoxicity [8,9,10].

Nebulized inhalation therapy has emerged as a promising alternative or adjunctive route for delivering antibiotics directly to the lung, thereby enhancing local concentrations while reducing systemic side effects [11,12]. Preclinical studies [13] demonstrate that inhaled PMB can reach ELF concentrations hundreds of times greater than those achieved via IV dosing. Recent clinical studies have also highlighted the potential benefits of nebulized antibiotics for treating MDR-GNB pneumonia [14,15,16]. Despite its potential advantages, current research on the pulmonary pharmacokinetics of PMB is very limited, making it difficult to develop evidence-based dosing regimens [17,18]. Furthermore, no studies have yet evaluated the pulmonary delivery capability of nebulized PMB injection in intensive care settings, particularly among mechanically ventilated patients, for whom nebulized drug administration faces complex delivery pathways and aerodynamic interference that may significantly reduce pulmonary delivery efficiency. In vitro studies have reported that less than 3% of the nominal nebulized dose reaches the patient’s lungs during mechanical ventilation, largely due to losses within the ventilator circuit [19,20,21].

Multiple factors influence the fate of an antibiotic aerosol as it passes through a ventilator circuit, including (i) the nebulizer’s physical principle and output rate; (ii) its location within the inspiratory limb; (iii) whether active humidification is used; (iv) the internal diameter and length of the artificial airway; and (v) patient-specific ventilatory parameters such as tidal volume, respiratory frequency, and duty cycle [22,23,24]. Without a quantitative understanding of how these factors collectively influence PMB pulmonary delivery and lung deposition, clinical dosing strategies remain largely empirical, potentially leading to suboptimal efficacy.

Hence, the primary objective of the present work was to generate an integrated, quantitative assessment of PMB aerosol pulmonary delivery during simulated invasive mechanical ventilation, focusing on the impact of nebulizer type, placement within the ventilator circuit, and the type of artificial airway. Secondary objectives were to delineate how these variables alter aerodynamic particle size distributions and to employ the multiple-path particle dosimetry (MPPD) model to predict regional lung deposition profiles. We hypothesized that nebulizer type, its placement, and the airway interface would each influence PMB aerosol delivery and lung deposition pattern.

To our knowledge, this is the first in vitro and in silico study to quantitatively evaluate the pulmonary delivery of nebulized polymyxin B injection during simulated adult invasive mechanical ventilation. By integrating detailed aerodynamic characterization with MPPD-based regional deposition predictions, this work provides actionable insights to guide optimization of aerosolized PMB therapy for mechanically ventilated patients with MDR-GNB infections.

## 2. Materials and Methods

### 2.1. Materials and Reagents

Polymyxin B sulfate for injection (500,000 IU/vial; Shanghai No. 1 Biochemical & Pharmaceutical Co., Ltd., Shanghai, China) was reconstituted in sterile 0.9% sodium chloride injection (500 mL; Hunan Kelun Pharmaceutical Co., Ltd., Yueyang, China) to yield a working solution of 5 mg/mL PMB. Ultrapure water, HPLC-grade methanol, and acetonitrile were obtained from Merck (Darmstadt, Germany). All filters, tubing, and adapters were pharmaceutical-grade components compatible with the nebulizers and ventilator circuit (See Supplementary Table S1 for details).

### 2.2. Experimental Apparatus

A Dräger Evita 4 mechanical ventilator (Dräger, Lübeck, Germany) with standard adult horizontal dual-limb circuit and heated humidifier (set to 37–40 °C outlet) simulated clinical ventilation. A breathing simulator (HRH-BRM2100, Beijing Huironghe Technology Co., Ltd., Beijing, China) reproduced spontaneous breathing waveforms. A Next-Generation Impactor (NGI; HRH-ZJQ160, Beijing Huironghe Technology Co., Ltd., Beijing, China) characterized aerodynamic particle distributions. Laser diffraction measurements were performed using a HELOS/INHALER module (Sympatec GmbH, Clausthal-Zellerfeld, Germany). PMB quantification was performed using HPLC (Shimadzu LC-2050, Kyoto, Japan) with a C18 column (150 × 4.6 mm, 5 µm) and UV detection at 215 nm (See Supplementary Table S2 for details).

### 2.3. Experimental Setup and Total Delivered Dose Evaluation

Two nebulizer types were evaluated: a vibrating mesh nebulizer (VMN; GUN-300vt, GENTEC, Shanghai, China) and a jet nebulizer (JN; TL-8, Bairui Medical Devices Co., Ltd., Changzhou, China). The JN was operated using compressed air from a PARI Junior BOY compressor, providing an output flow rate of 5.1 L/min. The ventilator or breathing simulator was configured to replicate the respiratory patterns of critically ill patients with pulmonary infection, based on typical ventilator settings extracted from the Medical Information Mart for Intensive Care-IV (MIMIC-IV, version 3.1, http://mimic.mit.edu) database [25] (tidal volume: 450 mL; respiratory frequency: 21 breaths/min; inspiratory-to-expiratory ratio: 1:3). Detailed data extraction procedures and parameter distributions are provided in the Supplementary Material (Figure S1 and Table S3).

**Figure 1:**
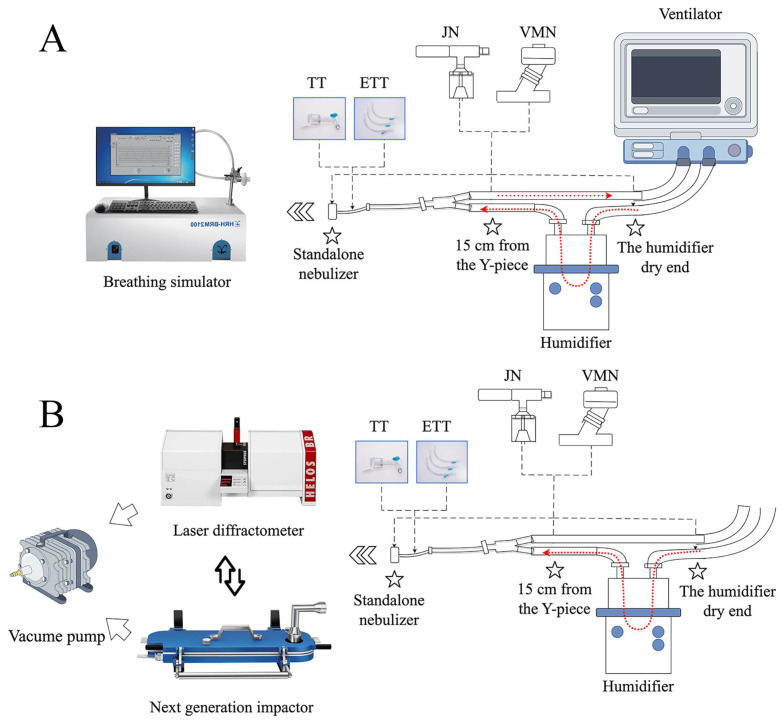
Experimental setups for aerosol delivery evaluation: (**A**) Schematic of the experimental configuration for the total delivered dose study. (**B**) Schematic of the in vitro pulmonary aerosol delivery performance study. Abbreviations: VMN, vibrating mesh nebulizer; JN, jet nebulizer; ETT, endotracheal tube; TT, tracheostomy tube.

Aerosol delivery was evaluated under two distinct experimental configurations: an invasive mechanical ventilation setup and a standalone nebulizer setup. The standalone nebulizer configuration served as a baseline, designed to simulate spontaneous breathing without the influence of a ventilator circuit. In this arrangement, the nebulizer outlet was connected directly to the collection filter at the breathing simulator’s inlet, thereby representing an idealized delivery scenario by eliminating aerosol losses within tubing. For the invasive mechanical ventilation scenarios, the nebulizer was integrated into a standard adult breathing circuit. Within this setup, two nebulizer placements (15 cm from the Y-piece vs. humidifier’s dry end) and two airway interfaces (a 7 mm internal diameter, 28 cm length ETT and a 7 mm internal diameter, 10 cm length TT) were tested, respectively. At the distal end of the artificial airway, a collection filter was connected via an adapter to capture the delivered dose, as illustrated in the schematic diagram (Figure 1). To ensure reproducibility, the lengths and angles of the circuit tubing were kept constant across all experimental conditions.

### 2.4. Sample Collection and Drug Quantification

In aerosol therapy, the delivered dose refers to the absolute mass of drug that reaches the airway outlet, typically measured via in vitro filters that simulate the entrance to the mouth or lungs. To facilitate comparisons across devices and configurations, the percentage of delivered dose normalizes this value relative to the initial nominal dose placed in the nebulizer reservoir, thereby reflecting pulmonary delivery efficiency.

In this study, a 65 mm polypropylene fiber filter was placed at the breathing simulator inlet to capture aerosol passing through the airway interface. Both VMN and JN were operated until no visible aerosol was produced, ensuring that the drug solution was fully nebulized and minimizing residual volumes (approximately 15 min for VMN and 30 min for JN). Upon completion, filters and all circuit segments (nebulizer reservoir, humidifier chamber, inspiratory/expiratory limb, artificial airway, extension tubes, and adapters) were individually recovered for mass balance analysis. To ensure complete drug recovery from the circuit, each component was meticulously disassembled. Depending on the part’s length and complexity, it was rinsed sequentially with 10–20 mL of ultrapure water. For tubing sections, the ends were sealed with adapters, and the tube was vigorously agitated to ensure the entire inner surface was thoroughly washed. Rinse solutions were filtered and brought to volume in calibrated flasks. PMB concentrations were determined by HPLC using an external standard calibration curve (r^2^ > 0.999).

### 2.5. In Vitro Evaluation of Pulmonary Aerosol Delivery Performance

#### 2.5.1. Next-Generation Impactor

Prior to testing, the NGI was conditioned at 5 °C in a refrigerated chamber for 90 min to minimize sample evaporation. The impactor was assembled per the manufacturer’s instructions and leak-tested to ≤2 kPa loss in 60 s. During NGI testing, the NGI inlet flow was held constant at 15 L·min^−1^ by an independent vacuum pump (operated continuously throughout each run) rather than being driven by the ventilator. The detailed instrument connection and experimental setup are illustrated in Figure 1. Nebulizers were loaded with 5 mL PMB solution. VMN ran for 15 min, JN for 25 min (ensuring complete dose nebulization). After aerosol generation, the pump ran for an additional 30 s to clear residual aerosol. Each NGI stage and the micro-orifice collector filter were rinsed, and the PMB mass was recovered. Data were used to compute mass median aerodynamic diameter (MMAD), geometric standard deviation, fine particle fraction (FPF), and stage-wise deposition.

#### 2.5.2. Laser Diffraction

Simultaneously, a parallel set of runs measured volume-based particle size distribution by laser diffraction (Figure 1). The laser module was connected at the distal end of the ventilator circuit or nebulizer nozzle via a custom adapter, with airflow controlled to 15 L/min by a vacuum pump like NGI. Key metrics included volume median diameter (X_50_), X_10_, X_90_, and percentage volume ≤ 5 µm. Three replicates per condition ensured reproducibility.

### 2.6. In Silico Regional Lung Deposition Modeling Using MPPD

Regional pulmonary deposition was predicted using the MPPD model (version 3.04, Applied Research Associates, https://www.ara.com/mppd, accessed on 1 December 2025), a widely-used deterministic model. The fundamental working principle of the MPPD model is to calculate particle deposition based on theoretical equations that describe the primary physical mechanisms of aerosol transport and deposition within the branching airways, including inertial impaction, gravitational sedimentation, and Brownian diffusion. The modeling process began by establishing the boundary conditions, for which we selected the stochastic Yeh/Schum five-lobe adult lung model, with a functional residual capacity of 3300 mL. We then configured the initial simulation parameters to mirror our experimental context, assuming a supine body orientation. The breathing scenario was defined with a tidal volume of 450 mL, a respiratory rate of 21 breaths/min, and an inspiratory-to-expiratory ratio of 1:3, simulating inhalation of PMB aerosols at a concentration of 5 g/cm^3^. The “Deposition Only” mode was selected for the simulation, excluding clearance processes. Input particle size distributions were derived directly from our NGI measurements, using the experimentally determined MMAD and geometric standard deviation (GSD) for each scenario. The model assumed a polydisperse aerosol and applied a diameter correction for particle density. For each experimental scenario, deposition fractions in the throat, tracheobronchial and alveolar regions were calculated and output.

### 2.7. Data Analysis

All experiments were performed in triplicate, and results are presented as mean ± standard deviation unless otherwise specified. All statistical analyses were conducted using R (Version 4.3.1, https://www.r-project.org/, accessed on 1 December 2025). A *p*-value of less than 0.05 was considered statistically significant for all tests.

The effects of nebulizer type, placement position, and artificial airway on delivered drug dose and laser diffraction particle size metrics under mechanical ventilation were analyzed using a three-way analysis of variance (ANOVA). For the APSD parameters, two-way ANOVAs were used to assess the impact of nebulizer type and placement position. When significant interactions were detected, a simple-effect analysis was conducted through pairwise comparisons of estimated marginal means with Tukey’s adjustment for multiple comparisons. Furthermore, the delivered dose and laser diffraction metrics in each mechanical ventilation scenario were compared against the standalone (baseline) condition for each nebulizer using a one-way ANOVA followed by Dunnett’s post hoc test. A direct comparison between the two nebulizers in the standalone condition was performed using an independent samples *t*-test. Prior to analysis, all ANOVA assumptions, including normality of residuals (Shapiro–Wilk test) and homogeneity of variances (Levene’s test), were verified.

## 3. Results

### 3.1. Aerosol Delivered Dose Under Spontaneous and Mechanical Ventilation

To evaluate the effects of these factors and their interactions, a three-way ANOVA was performed. The results showed highly significant main effects (all *p* < 0.0001, Supplementary Material Table S4) regarding nebulizer type, placement position, and artificial airway type. More importantly, all two-way interactions (Nebulizer × Position, Nebulizer × Airway, Position × Airway) and the three-way interaction (Nebulizer × Position × Airway) were statistically significant (all *p* < 0.014, Supplementary Material Table S4), indicating that the influence of any single factor on drug delivery depended on the levels of the other factors. Given the significant interactions, a simple-effect analysis was conducted to further elucidate these complex relationships.

**Figure 2:**
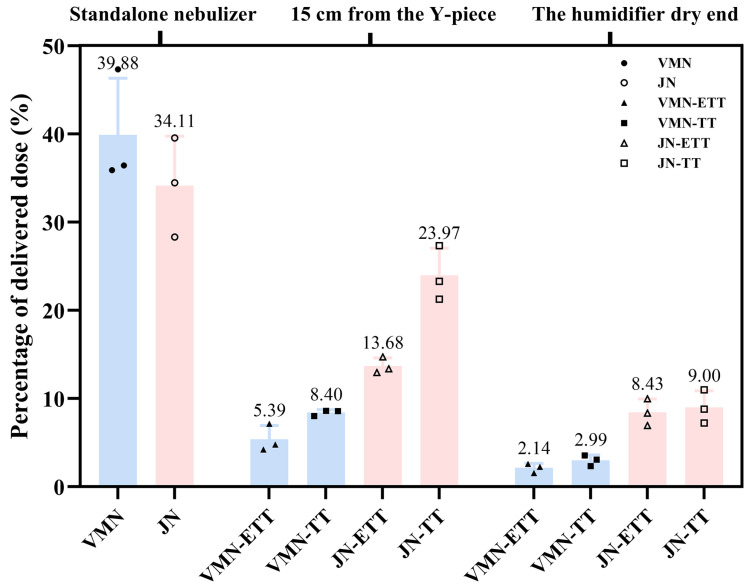
Percentage of delivered dose in each study scenario. The percentage was calculated based on an initial nebulized dose of 25 mg. Blue solid triangles and squares represent VMN, and red hollow triangles and squares represent JN.

In the in vitro delivery tests, measurable quantities of aerosolized polymyxin B reached the breathing simulator filter under both mechanical ventilation and standalone conditions. Delivery efficiency was significantly influenced by the nebulizer type, its placement, and the type of artificial airway. Under standalone nebulizer conditions, where drug loss is minimized due to the absence of ventilator tubing, bends, or humidification devices, the highest delivery efficiency was achieved. The mean delivered doses (Table 1) for the VMN and JN were 9.97 mg (39.9% of the 25 mg nominal dose) and 8.53 mg (34.1%), respectively. However, the difference in delivered dose between the two nebulizers was not statistically significant (*p* = 0.309). Compared with the baseline, the delivered dose was significantly lower across all mechanical ventilation scenarios (Dunnett’s test, all *p* ≤ 0.0085). The lowest dose was recorded for the VMN at the humidifier’s dry end, delivering only 2.1% and 3.0% of the nominal dose with an endotracheal tube and a tracheostomy tube, respectively (Figure 2).

The simple-effect analysis revealed the specific influences of each factor under mechanical ventilation conditions (Table 2). Regarding the effect of nebulizer type, the JN delivered a significantly higher drug dose than the VMN in all four mechanical ventilation combinations (all *p* ≤ 0.0002). Notably, when placed 15 cm from the Y-piece with a tracheostomy tube, the mean delivered dose for the JN was 5.99 mg (24.0%), which was 2.85-fold higher than the 2.10 mg (8.4%) delivered by the VMN. As for the effect of nebulizer position, placing the nebulizer closer to the patient (15 cm from the Y-piece) resulted in a significantly higher delivered dose for both nebulizers and airway types compared to placement at the dry end of the humidifier (all *p* ≤ 0.021). For instance, with the JN and an ETT, the delivery efficiency at 15 cm from the Y-piece position (13.7%) was significantly greater than at the humidifier position (8.4%) (*p* = 0.0008). The influence of artificial airway type was dependent on nebulizer position. When the nebulizer was placed at the 15 cm from Y-piece, the TT achieved higher delivery than the ETT for both the JN (*p* < 0.0001) and the VMN (*p* = 0.031). However, when the nebulizer was positioned at the humidifier, the difference between the two airway types was not statistically significant (*p* > 0.5).

### 3.2. Distribution of Drug Deposition in Ventilator Circuit Components

**Figure 3:**
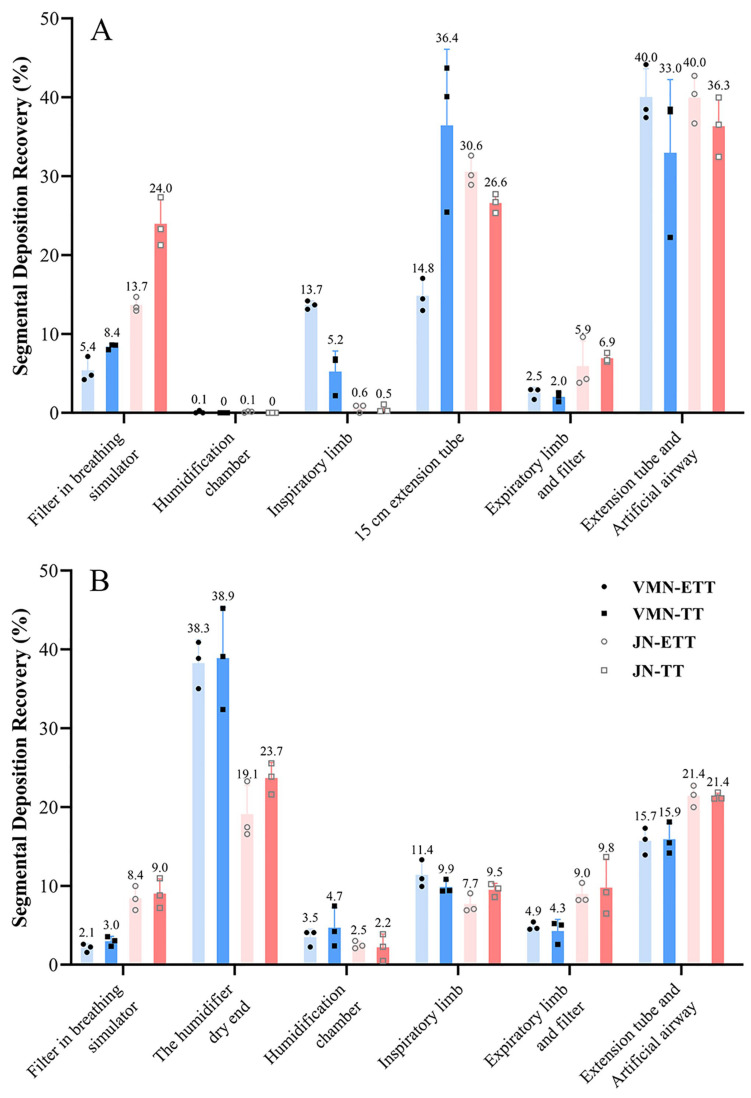
Segmental drug deposition in each study scenario: (**A**) 15 cm from the Y-piece; (**B**) the humidifier’s dry end. Panels (**A**,**B**) share the same legend, which is displayed within the panel (**B**). Blue solid circles and squares represent VMN, and red hollow circles and squares represent JN. Darker colors indicate TT, while lighter colors indicate ETT.

To clarify drug fate throughout the ventilator circuit, the mass of PMB recovered from each component—including nebulizer cup, inspiratory/expiratory limbs, humidifier, extension tubes, artificial airway, adapters, and filters—was quantified (Figure 3). Substantial drug losses were observed in ventilator circuits, particularly when the nebulizer was placed at the humidifier’s dry end. The main deposition sites were the artificial airway, the 15 cm extension tube, and the humidifier’s dry end. Notably, when the devices were evaluated at their respective aerosolization positions, JN still exhibited appreciable loss in the 15 cm extension tube—although the artificial airway remained the single largest sink—whereas the VMN showed its greatest loss at the humidifier’s dry end. These device-specific patterns highlight how nebulizer type and circuit configuration jointly determine the delivered dose.

### 3.3. Nebulizer Delivery Rate and Cumulative Filter Dose

Delivery rate data (Table 1) showed low initial drug accumulation on the filter during the first minute of nebulization under mechanical ventilation, likely due to system stabilization. The cumulative filter dose increased over time, particularly for the JN, which typically required 10–35 min to complete nebulization. Although the VMN delivered aerosol more rapidly, its total delivered dose was significantly reduced in ventilator settings compared to the standalone condition (Dunnett’s test, all *p* < 0.0001).

### 3.4. Aerodynamic Particle Size Distribution

A two-way ANOVA was subsequently applied to evaluate the effects of nebulizer type and placement position on the APSD parameters. The results demonstrated that both the main effects of nebulizer type and position, as well as their interaction, were statistically significant for all APSD parameters (all *p* ≤ 0.018, Supplementary Material Table S5). The simple-effects analysis further clarified the details of these interactions.

As shown in Table 3, the mechanical ventilation circuit significantly altered the aerosol characteristics of both nebulizers compared to the standalone condition. Overall, mechanical ventilation led to a smaller MMAD and GSD, along with an increased FPD and FPF, indicating a potential to improve pulmonary delivery performance.

Simple-effect analysis (Table 4) demonstrated that under mechanical ventilation (both at the Y-piece and humidifier positions), the JN produced aerosols with a significantly smaller (i.e., more optimal) MMAD and a higher FPF than the VMN (all *p* < 0.0001). However, under standalone conditions, the opposite was observed: the JN’s MMAD was significantly larger than the VMN’s (4.53 μm vs. 4.25 μm, *p* = 0.0009), and its FPF was significantly lower (55.51% vs. 60.14%, *p* = 0.0018). As for the effect of nebulizer position, all APSD parameters changed significantly between mechanical ventilation and standalone conditions (*p* = 0.0001). For VMN, most APSD parameters did not differ significantly between the Y-piece and humidifier positions, but the FPF was significantly higher at the Y-piece position (*p* = 0.0429). For the JN, placement at the Y-piece produced a significantly smaller MMAD (2.73 μm vs. 2.93 μm, *p* = 0.0171) and a larger GSD (1.65 vs. 1.54, *p* = 0.0006) compared to the humidifier position.

**Figure 4:**
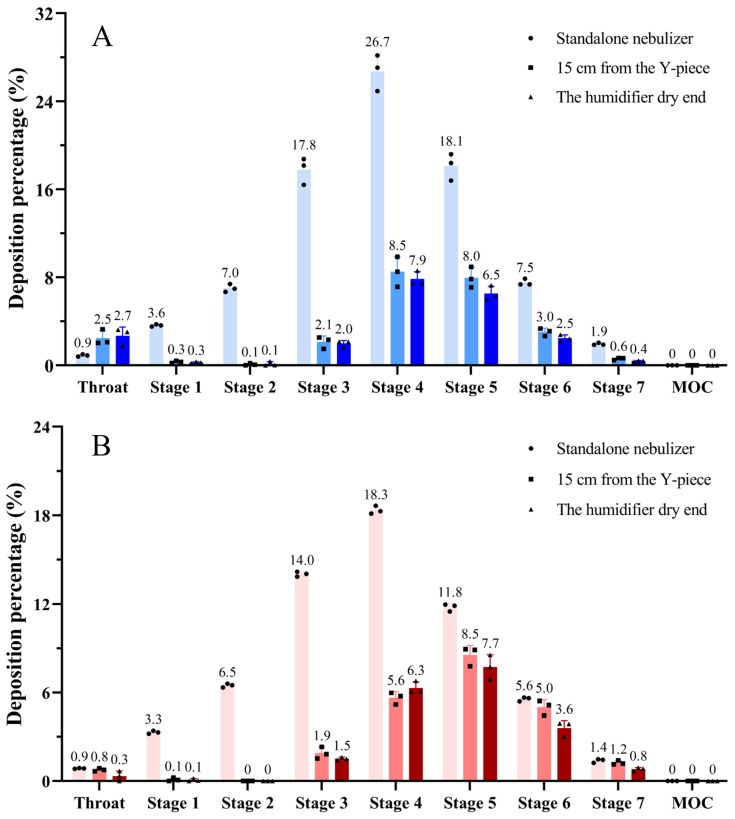
Percentage of drug deposition on each stage of NGI: (**A**) the vibrating mesh nebulizer; (**B**) the jet nebulizer. Light, medium, and dark colors represent the standalone nebulizer, 15 cm from the Y-piece, and the humidifier’s dry end.

NGI deposition analysis (Figure 4) indicated that, for both VMN and JN nebulizers, the majority of drug deposition under a standalone nebulizer occurred at NGI stage 4 and stage 3, indicating a mass median aerodynamic diameter in the small-to-medium particle size range. When the nebulizer was incorporated into a mechanical ventilation circuit, the proportion of deposition associated with larger particle stages decreased, while deposition in the fine particle regions (NGI stages 4 and beyond) increased.

### 3.5. Laser Diffraction Particle Size Analysis

This study utilized laser diffraction analysis to evaluate aerosol particle size distributions from VMN and JN nebulizers (Table 5). The results generally aligned with NGI, though numerical differences were observed. Overall, VMN produced aerosols with smaller particle sizes and higher proportions of particles ≤5 μm, particularly under standalone nebulizer conditions and at 15 cm from the Y-piece. At the humidifier’s dry end, particle sizes from both nebulizers were comparable, likely due to increased aerosol particle hydration induced by humidification.

Introducing the nebulizers into mechanical ventilation circuits shifted particle size distribution peaks toward smaller sizes, resulting in narrower and sharper distributions. This shift suggests that ventilator circuits effectively filter out larger aerosol particles, thereby enhancing the delivery of finer particles to the peripheral airways and alveolar regions. Notably, compared to endotracheal intubation, the tracheostomy circuit is shorter and oriented more vertically, resulting in more pronounced instantaneous changes in airflow direction. This enhanced aerosol particle impaction, consequently reducing the mean aerosol particle size and increasing the fine particle fraction under tracheostomy conditions (Detailed statistical analyses are provided in Supplementary Tables S6–S9).

### 3.6. MPPD Simulation of Regional Lung Deposition

Regional aerosol deposition within the bronchial and alveolar regions was predicted using the MPPD software under six different delivery scenarios. As shown in Table 6, pulmonary deposition fractions were higher with the VMN nebulizer compared to the JN device in all situations. In the absence of ventilator circuits, both nebulizers demonstrated substantial aerosol deposition in the throat region, accounting for approximately 26.88% (VMN) and 29.61% (JN), while bronchial (11.16% VMN, 11.47% JN) and alveolar depositions (13.80% VMN, 12.94% JN) remained relatively low.

In contrast, upon connection to the ventilator circuits, aerosol deposition in the throat region was eliminated, while alveolar deposition increased. Specifically, alveolar deposition increased to 26.79–27.51% (VMN) and 23.32–24.94% (JN)—approximately double the baseline values observed in standalone configurations. Tracheobronchial deposition showed only minor increases under ventilation (ranging from 2.26–5.05% across conditions). Notably, predicted regional deposition fractions exhibited minimal differences between nebulizer types or placement positions within the circuit.

**Figure 5:**
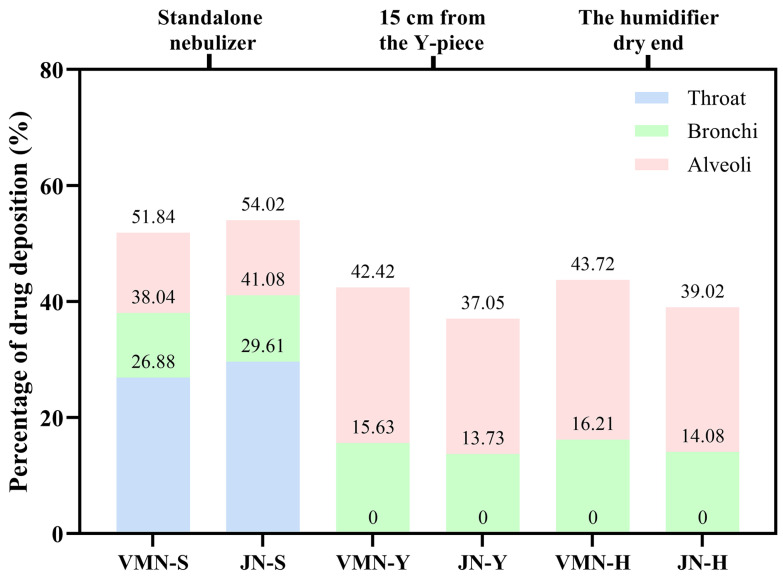
MPPD-predicted regional aerosol deposition fractions in standalone nebulization and mechanical ventilation scenarios. The data presented above are unadjusted; the actual lung deposition fractions were obtained following adjustment for delivered dose. VMN-S and JN-S (standalone nebulizer); VMN-Y and JN-Y (nebulizer placed 15 cm from the Y-piece); VMN-H and JN-H (nebulizer placed at the dry end of the heated humidifier).

This comparative analysis (Figure 5) underscores a key trade-off associated with mechanical ventilation circuits: although the total aerosol mass delivered to the lungs is substantially reduced, the circuit effectively functions as a filter, removing larger droplets through impaction and condensation within the tubing and heated humidifier. The resulting finer aerosol promotes deeper lung penetration and enhanced alveolar deposition. Overall, these MPPD predictions align closely with the experimental observations of altered aerosol APSD characteristics during mechanical ventilation.

## 4. Discussion

In this study, an integrated in vitro simulation model was developed to systematically evaluate the aerosol delivery characteristics of PMB under invasive mechanical ventilation and standalone nebulizer conditions. Our findings highlight several clinically relevant factors crucial for optimizing nebulized antibiotic therapy in ventilated patients with MDR-GNB pulmonary infections.

Consistent with previous reports [21,26], the standalone nebulizer yielded significantly higher aerosol delivered dose compared with all mechanical ventilation scenarios (Dunnett’s test, *p* ≤ 0.0085 for all comparisons). In this standalone setting, the VMN, with its higher aerosol output rate and superior aerosol characteristics (lower MMAD and higher FPF), as also reported in the literature [27,28,29], showed a trend toward slightly higher drug delivery compared to the JN, although this difference did not reach statistical significance (*p* = 0.309). This observation supports the reliability of this in vitro model in mimicking the performance of nebulizers under spontaneous breathing conditions.

However, under mechanical ventilation, the drug delivered dose declines markedly. Regardless of whether VMN or JN is used, the introduction of a mechanical ventilation circuit results in substantial drug loss during transmission from the nebulizer to the simulated lung, with the delivered dose significantly lower than under a standalone nebulizer. Specifically, when the nebulizer was placed at the dry end of the heated humidifier, the filter collection rate for VMN fell below 3%, and although slightly higher for JN, the overall efficiency remained low. Further analysis revealed considerable drug deposition at various circuit segments, including the artificial airway, inspiratory limb (with the 15 cm extension tube), and within the heated humidifier, consistent with previous studies [22,30,31,32]. For example, a previous systematic review [22] indicated that circuit antibiotic losses ranged from 34% to 78%, comprising 10–44% retention in tubing, 1–27% loss in the artificial airway or trachea, and 7–22% loss in the expiratory limb and filter. These findings suggest that the combined effects of elongated, curved circuit architecture and the heated humidification environment impose significant barriers to aerosol transmission [33]. On one hand, abrupt changes in airflow direction at bends and connectors promote inertial impaction. On the other hand, the high temperature and humidity of the heated humidifier facilitate droplet coalescence and re-deposition. Overall, the drug recovered from circuit components typically exceeds that collected on the filter, resulting in a marked reduction in the effective dose delivered to the pulmonary.

Among most ventilated scenarios (with low or without basic airflow), placement of the nebulizer 15 cm from the Y-piece significantly improved aerosol delivered dose compared to placement at the humidifier’s dry end. As confirmed by our simple-effect analysis, this finding holds true for both nebulizer types and across both artificial airways (all *p* ≤ 0.021), aligning with prior studies [34,35,36]. The longer circuit and humidifying effects at the humidifier’s dry end resulted in greater drug loss, as evidenced by a substantial increase in uncollected drug. Positioning nebulizers closer to the patient in the inspiratory limb reduces the likelihood of aerosol particle impaction and condensation within upstream tubing, thereby enhancing the fraction of aerosol delivered to the pulmonary.

Our comparative evaluation of VMN and JN nebulizers under prolonged nebulization conditions demonstrated important insights regarding nebulizer performance characteristics under ventilated conditions. Our results showed that all APSD parameters changed significantly between mechanical ventilation and standalone conditions (*p* = 0.0001). An important finding is that the superiority of one nebulizer type over another is context-dependent, and the assumptions regarding which is better are not universally applicable. While the VMN exhibited superior aerosol characteristics in the standalone setting, the performance reversed under mechanical ventilation. This apparent paradox may stem from several factors, including the interaction between nebulizer output rate, the ventilator settings, and the measurement endpoint. The JN achieved significantly higher delivered doses and FPF than the VMN, along with lower MMAD across all four ventilated scenarios (all *p* ≤ 0.0002).

Two main factors may explain these findings. First, the rapid aerosolization rate of the VMN, while advantageous in standalone conditions, may have become a liability under the low inspiratory flow rate and prolonged expiratory phase used in our mechanical ventilation model. Specifically, the VMN’s high output rate may have exceeded the carrying capacity of the ventilator’s inspiratory flow, leading to excessive aerosol impaction and condensation within the circuit tubing, especially at connection points. This phenomenon has been reported in prior studies [22,37,38] where rapid aerosol generation in a confined space leads to substantial drug loss. Second, our findings highlight a critical distinction between delivery rate and cumulative delivery efficiency, which depends on nebulizing to completion. It is noteworthy that some influential early studies supporting VMN’s superiority may have inadvertently disadvantaged the JN by using fixed, and potentially incomplete, nebulization times rather than ensuring full dose delivery. A short runtime naturally favors a faster nebulizer when assessing delivery rate, but it may not reflect the total achievable dose. Inadequate nebulization has been shown to leave as much as 50–65% of the drug in the JN reservoir and T-piece [29,39,40,41,42,43,44]. In contrast, our study protocol defined completion as the absence of visible aerosol for 15 s, ensuring that the full potential of each device was evaluated. Under these conditions, the JN’s slower but stable and continuous output demonstrated a clear cumulative advantage. Notably, the finding that JN outperformed VMN under mechanical ventilation may be limited to the specific conditions of this study, including the tested nebulizer models, specific ventilation parameters, heated humidifier setup, and complete nebulization protocol. Generalization to other devices, ventilator settings, or clinical contexts requires further validation.

Both NGI and laser diffraction measurements consistently demonstrated shifts toward smaller aerosol particle sizes and higher fine particle fractions when nebulizers were integrated into ventilator circuits. In the NGI stage-wise deposition profiles, this circuit-induced refinement manifested as reduced or absent drug mass on stages 1–4, with the overall distribution trending toward smaller sizes. This shift to finer aerosols is consistent with in vitro observations reported by Liu et al. [45] in an adult mechanical ventilation model for colistin delivery. They noted the disappearance of upper-stage deposition in the Andersen cascade impactor (with drug detected only on stages 3–7), a substantial increase in fine particle fraction distal to the endotracheal tube (FPF > 87–95%), and a reduction in GSD to near-monodisperse levels (about 1.4 μm). Computational simulations using the MPPD software further provided additional insights into regional lung deposition patterns under different nebulization conditions. Based on the predictions of the MPPD model, this shift toward smaller aerosol particle sizes substantially increases the proportion of drug deposition in the peripheral regions of the pulmonary—by approximately twofold. Although quantitative modeling of pulmonary deposition for nebulized polymyxin B or colistin using the MPPD model is currently scarce, our findings are consistent with previous systematic reviews. Dugernier et al. [22], in their comprehensive review of 26 clinical studies and 10 experimental studies, reported that whole-lung deposition with nebulizers is typically less than 20% of the nominal dose, with values commonly ranging from 1% to 16% in scintigraphic and pharmacokinetic studies employing conventional administration techniques. In our study, the total lung deposition fractions after adjustment for delivered dose ranged from 0.94% to 5.07% under ventilated conditions. These results further corroborate the concept that ventilator tubing effectively filters out larger aerosol droplets through inertial impaction, resulting in finer aerosol fractions reaching the patient. However, this benefit must be balanced against the substantial overall decrease in total aerosol mass delivered due to upstream losses.

This study has several limitations. First, comprehensive aerodynamic particle size assessments using the NGI were performed only for the endotracheal tube conditions. While these findings were corroborated by laser diffraction data across all groups, the absence of direct NGI measurements for the tracheostomy scenarios limits a full characterization of aerosol behavior in those specific setups. Second, the evaluation was confined to a single commercial model of each nebulizer type and based on a limited number of replicates (*n* = 3) for each scenario. Given the known performance variability among different nebulizer brands [46] and small sample size, the quantitative results should be interpreted as specific to the tested conditions, and caution is advised when generalizing them. These factors highlight the need for future studies involving a broader range of devices to confirm the generalizability of our findings. The absence of in vivo experiments is also a limitation; this study focused on controlled in vitro and in silico modeling to isolate variables. In vivo evaluation of PMB is complicated by pulmonary pharmacokinetics and was beyond the scope and current facilities, though it represents an important future direction.

## 5. Conclusions

In conclusion, this study provides the first detailed quantitative in vitro and in silico assessment of PMB aerosol delivery during invasive mechanical ventilation. We identified critical modifiable factors—including nebulizer type, placement within the ventilator circuit, and artificial airway—that significantly alter aerosol pulmonary delivery and deposition. Standalone nebulization achieved 34–40% delivered dose, reduced to 2–24% in ventilation, depending on configuration. Circuit integration filtered larger particles, yielding smaller MMAD and higher FPF, and finally doubling MPPD-predicted alveolar deposition (from 13–14% in standalone to 23–28% in ventilation scenarios). These findings offer practical guidelines to enhance the clinical effectiveness of aerosolized PMB therapy in critically ill, mechanically ventilated patients.

## Figures and Tables

**Table 1:** The delivered dose captured by the filter at each time interval.

Position	Nebulizer	Artificial Airway	0–1 min	1–5 min	5–10 min	10 min–End	Total (Mean ± SD)
Standalone nebulizer	VMN	-	0.55	3.4	4.22	1.80	9.97 ± 1.61
	JN	-	0.01	0.93	2.8	4.84	8.53 ± 1.41
15 cm from the Y-piece	VMN	ETT	0.00	0.49	0.72	0.13	1.35 ± 0.39
		TT	0.00	0.60	1.20	0.30	2.10 ± 0.09
	JN	ETT	0.00	0.24	0.90	2.28	3.42 ± 0.23
		TT	0.00	0.48	1.43	4.08	5.99 ± 0.77
The humidifier’s dry end	VMN	ETT	0.00	0.19	0.35	0.00	0.54 ± 0.13
		TT	0.00	0.23	0.51	0.00	0.75 ± 0.16
	JN	ETT	0.00	0.05	0.50	1.56	2.11 ± 0.38
		TT	0.00	0.02	0.63	1.60	2.25 ± 0.47

Note: Data are presented as mean values (*n* = 3, mg). Abbreviations: SD, standard deviation.

**Table 2:** Simple-effect analysis and baseline comparisons of total delivered dose under different nebulization scenarios.

Comparison and Contrast	Mean Difference (SE)	Test Statistic (df)	*p*-Value
Part A. Pairwise comparisons within mechanical ventilation (from three-way ANOVA)			
1. Nebulizer effect (JN vs. VMN)			
At the humidifier dry end, ETT	1.57 (0.32)	t (16) = 4.95	0.0001
At the humidifier’s dry end, TT	1.50 (0.32)	t (16) = 4.73	0.0002
At Y-piece (15 cm), ETT	2.07 (0.32)	t (16) = 6.53	<0.0001
At Y-piece (15 cm), TT	3.89 (0.32)	t (16) = 12.25	<0.0001
2. Position effect (Humidifier dry end vs. Y-piece 15 cm)			
For JN, ETT	−1.31 (0.32)	t (16) = −4.13	0.0008
For JN, TT	−3.74 (0.32)	t (16) = −11.78	<0.0001
For VMN, ETT	−0.81 (0.32)	t (16) = −2.55	0.021
For VMN, TT	−1.35 (0.32)	t (16) = −4.26	0.0006
3. Airway effect (ETT vs. TT)			
For JN, Y-piece (15 cm)	−2.57 (0.32)	t (16) = −8.09	<0.0001
For JN, Humidifier dry end	−0.142 (0.32)	t (16) = −0.446	0.662
For VMN, Y-piece (15 cm)	−0.75 (0.32)	t (16) = −2.37	0.031
For VMN, Humidifier dry end	−0.211 (0.32)	t (16) = −0.665	0.516
Part B. Mechanical ventilation vs. nebulizer baseline (Dunnett’s test)			
1. VMN nebulizer			
Humidifier dry end + ETT vs. standalone	−9.43 (0.61)	t (10) = −15.47	<0.0001
Y-piece (15 cm) + ETT vs. standalone	−8.62 (0.61)	t (10) = −14.14	<0.0001
Humidifier dry end + TT vs. standalone	−9.22 (0.61)	t (10) = −15.12	<0.0001
Y-piece (15 cm) + TT vs. standalone	−7.87 (0.61)	t (10) = −12.90	<0.0001
2. JN nebulizer			
Humidifier dry end + ETT vs. standalone	−6.42 (0.63)	t (10) = −10.16	<0.0001
Y-piece (15 cm) + ETT vs. standalone	−5.11 (0.63)	t (10) = −8.08	<0.0001
Humidifier dry end + TT vs. standalone	−6.28 (0.63)	t (10) = −9.94	<0.0001
Y-piece (15 cm) + TT vs. standalone	−2.54 (0.63)	t (10) = −4.01	0.0085
Part C. Direct comparison under a standalone nebulizer (independent *t*-test)			
JN vs. VMN	—	t (3.93) = −1.17	0.309

Abbreviations: SE, standard error; df, degrees of freedom; ANOVA, analysis of variance.

**Table 3:** Calculated APSD parameters in the endotracheal-tube condition.

Parameters	Standalone Nebulizer		15 cm from the Y-Piece		The Humidifier’s Dry End	
	**VMN**	**JN**	**VMN**	**JN**	**VMN**	**JN**
Nebulized dose (mg)	25.00					
Calc. Delivered (mg)	20.92 ± 1.07	15.45 ± 0.22	6.28 ± 0.90	5.80 ± 0.50	5.58 ± 0.61	5.09 ± 0.511
FPD (mg)	12.58 ± 0.69	8.58 ± 0.16	4.81 ± 0.55	4.97 ± 0.39	4.11 ± 0.39	4.46 ± 0.431
FPF (%)	60.14 ± 0.28	55.51 ± 0.32	76.86 ± 2.78	85.69 ± 1.28	73.66 ± 1.50	87.67 ± 0.581
MMAD (μm)	4.25 ± 0.01	4.53 ± 0.02	3.25 ± 0.15	2.73 ± 0.05	3.37 ± 0.05	2.93 ± 0.071
GSD	1.86 ± 0.03	1.92 ± 0.01	1.51 ± 0.01	1.65 ± 0.04	1.49 ± 0.02	1.54 ± 0.011

Note: Data are presented as mean ± standard deviation (*n* = 3). Detailed statistical comparisons are shown in Table 4. Abbreviations: Calc. Delivered, calculated delivered dose (refers to the amount of drug that was captured by the NGI); FPD, fine particle dose; FPF, fine particle fraction; MMAD, mass median aerodynamic diameter; GSD, geometric standard deviation.

**Table 4:** Simple-effect analysis of nebulizer type and position on aerodynamic parameters.

Comparison	Calc. Delivered	FPD	FPF	MMAD	GSD
1. Effect of Position within each Nebulizer					
For VMN, Standalone vs. Y-piece (15 cm)	<0.0001	<0.0001	<0.0001	<0.0001	<0.0001
For VMN, Standalone vs. Humidifier dry end	<0.0001	<0.0001	<0.0001	<0.0001	<0.0001
For VMN, Y-piece (15 cm) vs. Humidifier dry end	0.4572	0.1935	0.0429	0.1547	0.6674
For JN, Standalone vs. Y-piece (15 cm)	<0.0001	<0.0001	<0.0001	<0.0001	<0.0001
For JN, Standalone vs. Humidifier dry end	<0.0001	<0.0001	<0.0001	<0.0001	<0.0001
For JN, Y-piece (15 cm) vs. Humidifier dry end	0.4476	0.416	0.2435	0.0171	0.0006
2. Effect of Nebulizer type at each Position					
At Standalone	<0.0001	<0.0001	0.0018	0.0009	0.0146
At Y-piece (15 cm)	0.4184	0.6865	<0.0001	<0.0001	<0.0001
At Humidifier dry end	0.4089	0.3564	<0.0001	<0.0001	0.0307

**Table 5:** Laser diffraction analysis of aerosol particle size distribution in each study scenario.

Position	Nebulizer	Artificial Airway	X_10_ (µm)	X_50_ (µm)	X_90_ (µm)	Proportion of Particles Size ≤5 µm (%)
Standalone nebulizer	VMN	-	0.93 ± 0.11	3.42 ± 0.04	7.46 ± 0.21	71.54 ± 0.65
	JN	-	1.49 ± 0.07	4.78 ± 0.11	10.87 ± 0.34	52.42 ± 1.28
15 cm from the Y-piece	VMN	ETT	0.87 ± 0.08	3.07 ± 0.01	5.84 ± 0.11	83.52 ± 0.73
		TT	0.86 ± 0.03	2.95 ± 0.03	5.14 ± 0.07	88.44 ± 0.75
	JN	ETT	1.41 ± 0.05	3.71 ± 0.07	7.21 ± 0.15	69.27 ± 1.57
		TT	1.23 ± 0.04	3.44 ± 0.10	6.41 ± 0.17	75.83 ± 1.63
The humidifier’s dry end	VMN	ETT	1.11 ± 0.08	3.38 ± 0.06	5.94 ± 0.19	80.33 ± 2.05
		TT	1.04 ± 0.06	3.36 ± 0.10	5.84 ± 0.17	81.41 ± 2.31
	JN	ETT	1.36 ± 0.10	3.33 ± 0.09	6.21 ± 0.14	78.61 ± 1.10
		TT	1.22 ± 0.05	3.23 ± 0.05	5.93 ± 0.05	81.48 ± 0.54

Note: Data are presented as mean ± standard deviation (*n* = 9). Abbreviations: X_10_, X_50_, X_90_: particle diameter at the 10th, 50th, and 90th percentile by volume, respectively.

**Table 6:** MPPD-predicted drug deposition in endotracheal-tube conditions (*n* = 3).

Deposition Site	Standalone Nebulizer		15 cm from the Y-Piece		The Humidifier’s Dry End	
	VMN	JN	VMN	JN	VMN	JN
Throat (%)	26.88	29.61	0.00	0.00	0.00	0.00
Bronchi (%)	11.16	11.47	15.63	13.73	16.21	14.08
Alveoli (%)	13.80	12.94	26.79	23.32	27.51	24.94

Note: The data presented above are unadjusted; the actual lung deposition fractions were obtained following adjustment for delivered dose. Deposition regions are defined as Throat (extrathoracic), Bronchi (tracheobronchial), and Alveoli (alveolar).

## Data Availability

The original contributions presented in this study are included in the article/Supplementary Material. Further inquiries can be directed to the corresponding authors.

## Supplementary Materials

The following supporting information can be downloaded at: https://www.mdpi.com/article/10.3390/pharmaceutics18010058/s1, Figure S1. Flowchart of patient extraction from MIMIC-IV database; Table S1. Materials and reagents used in the study; Table S2. Experimental apparatus and their purposes in the study; Table S3. Respiratory parameters of critically ill patients with pulmonary infection from the MIMIC-IV database; Table S4. Results of three-way ANOVA for delivered dose under mechanical ventilation; Table S5. Results of two-way ANOVA for aerodynamic particle size distribution parameters; Table S6. Three-way ANOVA results for median particle size (X50) from laser diffraction analysis; Table S7. Simple-effect analyses and baseline comparisons for median particle size (X50) from laser diffraction; Table S8. Statistical analysis of the proportion of particles ≤ 5 µm determined by laser diffraction; Table S9. Simple-effect analysis and baseline comparisons for the proportion of particles ≤ 5 µm determined by laser diffraction.

## Abbreviations

ANOVA	Analysis of Variance
APSD	Aerodynamic Particle Size Distribution
ELF	Epithelial Lining Fluid
ETT	Endotracheal Tube
FPF	Fine Particle Fraction
FPD	Fine Particle Dose
GSD	Geometric Standard Deviation
HPLC	High-Performance Liquid Chromatography
JN	Jet Nebulizer
MDR-GNB	Multidrug-Resistant Gram-Negative Bacteria
MIMIC-IV	Medical Information Mart for Intensive Care-IV
MMAD	Mass Median Aerodynamic Diameter
MPPD	Multiple-Path Particle Dosimetry
NGI	Next-Generation Impactor
PMB	Polymyxin B
TT	Tracheostomy Tube
VMN	Vibrating Mesh Nebulizer

## References

[1] Assefa M. (2022). Multi-drug resistant gram-negative bacterial pneumonia: Etiology, risk factors, and drug resistance patterns. Pneumonia.

[2] Almyroudi M.P., Chang A., Andrianopoulos I., Papathanakos G., Mehta R., Paramythiotou E., Koulenti D. (2024). Novel Antibiotics for Gram-Negative Nosocomial Pneumonia. Antibiotics.

[3] Bassetti M., Welte T., Wunderink R.G. (2016). Treatment of Gram-negative pneumonia in the critical care setting: Is the beta-lactam antibiotic backbone broken beyond repair?. Crit. Care.

[4] Sati H., Carrara E., Savoldi A., Hansen P., Garlasco J., Campagnaro E., Boccia S., Castillo-Polo J.A., Magrini E., Garcia-Vello P. (2025). The WHO Bacterial Priority Pathogens List 2024: A prioritisation study to guide research, development, and public health strategies against antimicrobial resistance. Lancet Infect. Dis..

[5] Zhang X., Qi S., Duan X., Han B., Zhang S., Liu S., Wang H., Zhang H., Sun T. (2021). Clinical outcomes and safety of polymyxin B in the treatment of carbapenem-resistant Gram-negative bacterial infections: A real-world multicenter study. J. Transl. Med..

[6] Eslami M., Safaripour A., Banihashemian S.Z., Nikjoo Niaragh S., Hemmati M.A., Shojaeian A., Fakhariyan S., Rabbani A., Oksenych V. (2025). Innovative Antibiotic Therapies for Carbapenem-Resistant Gram-Negative Bacterial Infections: Clinical Efficacy, Safety, and Comparative Studies. Microorganisms.

[7] Nang S.C., Azad M.A.K., Velkov T., Zhou Q.T., Li J. (2021). Rescuing the Last-Line Polymyxins: Achievements and Challenges. Pharmacol. Rev..

[8] Shi R., Fu Y., Gan Y., Wu D., Zhou S., Huang M. (2023). Use of polymyxin B with different administration methods in the critically ill patients with ventilation associated pneumonia: A single-center experience. Front. Pharmacol..

[9] Lakota E.A., Landersdorfer C.B., Nation R.L., Li J., Kaye K.S., Rao G.G., Forrest A. (2018). Personalizing Polymyxin B Dosing Using an Adaptive Feedback Control Algorithm. Antimicrob. Agents Chemother..

[10] Landersdorfer C.B., Wang J., Wirth V., Chen K., Kaye K.S., Tsuji B.T., Li J., Nation R.L. (2018). Pharmacokinetics/pharmacodynamics of systemically administered polymyxin B against Klebsiella pneumoniae in mouse thigh and lung infection models. J. Antimicrob. Chemother..

[11] Dallal Bashi Y.H., Mairs R., Murtadha R., Kett V. (2025). Pulmonary Delivery of Antibiotics to the Lungs: Current State and Future Prospects. Pharmaceutics.

[12] Rello J., Rouby J.J., Sole-Lleonart C., Chastre J., Blot S., Luyt C.E., Riera J., Vos M.C., Monsel A., Dhanani J. (2017). Key considerations on nebulization of antimicrobial agents to mechanically ventilated patients. Clin. Microbiol. Infect..

[13] Lin Y.W., Zhou Q., Onufrak N.J., Wirth V., Chen K., Wang J., Forrest A., Chan H.K., Li J. (2017). Aerosolized Polymyxin B for Treatment of Respiratory Tract Infections: Determination of Pharmacokinetic-Pharmacodynamic Indices for Aerosolized Polymyxin B against Pseudomonas aeruginosa in a Mouse Lung Infection Model. Antimicrob. Agents Chemother..

[14] Zhou Y., Wang G., Zhao Y., Chen W., Chen X., Qiu Y., Liu Y., Wu S., Guan J., Chang P. (2024). Efficacy and safety of different polymyxin-containing regimens for the treatment of pneumonia caused by multidrug-resistant gram-negative bacteria: A systematic review and network meta-analysis. Crit. Care.

[15] Cheng Y., Zhou L., Wang D., Li X., Lin R., Chen J., Tu F., Lin Y., Wu W., Liu M. (2025). Inhaled alone versus inhaled plus intravenous polymyxin B for the treatment of pneumonia due to carbapenem-resistant gram-negative bacteria: A prospective randomized controlled trial. Int. J. Antimicrob. Agents.

[16] Wu Z., Zhang S., Cao Y., Wang Q., Sun K., Zheng X. (2023). Comparison of the clinical efficacy and toxicity of nebulized polymyxin monotherapy and combined intravenous and nebulized polymyxin for the treatment of ventilator-associated pneumonia caused by carbapenem-resistant gram-negative bacteria: A retrospective cohort study. Front. Pharmacol..

[17] Tsuji B.T., Pogue J.M., Zavascki A.P., Paul M., Daikos G.L., Forrest A., Giacobbe D.R., Viscoli C., Giamarellou H., Karaiskos I. (2019). International Consensus Guidelines for the Optimal Use of the Polymyxins: Endorsed by the American College of Clinical Pharmacy (ACCP), European Society of Clinical Microbiology and Infectious Diseases (ESCMID), Infectious Diseases Society of America (IDSA), International Society for Anti-infective Pharmacology (ISAP), Society of Critical Care Medicine (SCCM), and Society of Infectious Diseases Pharmacists (SIDP). Pharmacotherapy.

[18] Li X., Zhou L., Wang D., Wu Q., Huang X., Zhang H., Wu W., Liu M., Wu X., Qiu H. (2025). Population pharmacokinetics study on nebulized and intravenous administration of polymyxin B in patients with pneumonia caused by multidrug-resistant gram-negative bacteria. Antimicrob. Agents Chemother..

[19] Harvey C.J., O’Doherty M.J., Page C.J., Thomas S.H., Nunan T.O., Treacher D.F. (1997). Comparison of jet and ultrasonic nebulizer pulmonary aerosol deposition during mechanical ventilation. Eur. Respir. J..

[20] MacIntyre N.R., Silver R.M., Miller C.W., Schuler F., Coleman R.E. (1985). Aerosol delivery in intubated, mechanically ventilated patients. Crit. Care Med..

[21] Lin H.L., Fink J.B., Ge H. (2021). Aerosol delivery via invasive ventilation: A narrative review. Ann. Transl. Med..

[22] Dugernier J., Ehrmann S., Sottiaux T., Roeseler J., Wittebole X., Dugernier T., Jamar F., Laterre P.F., Reychler G. (2017). Aerosol delivery during invasive mechanical ventilation: A systematic review. Crit. Care.

[23] Desgrouas M., Ehrmann S. (2021). Inhaled antibiotics during mechanical ventilation-why it will work. Ann. Transl. Med..

[24] Dhand R. (2008). Aerosol delivery during mechanical ventilation: From basic techniques to new devices. J. Aerosol Med. Pulm. Drug Deliv..

[25] Johnson A.E.W., Bulgarelli L., Shen L., Gayles A., Shammout A., Horng S., Pollard T.J., Hao S., Moody B., Gow B. (2023). Author Correction: MIMIC-IV, a freely accessible electronic health record dataset. Sci. Data.

[26] Dhand R. (2017). How Should Aerosols Be Delivered During Invasive Mechanical Ventilation?. Respir. Care.

[27] Bateman R.M., Sharpe M.D., Jagger J.E., Ellis C.G., Solé-Violán J., López-Rodríguez M., Herrera-Ramos E., Ruíz-Hernández J., Borderías L., Horcajada J. (2016). 36th International Symposium on Intensive Care and Emergency Medicine: Brussels, Belgium. 15–18 March 2016. Crit. Care.

[28] Mac Giolla Eain M., O’Sullivan A., Joyce M., MacLoughlin R. (2021). In vitro evaluation of disposable transport ventilators with combination aerosol therapy. BMJ Open Respir. Res..

[29] Naughton P.J., Joyce M., Mac Giolla Eain M., O’Sullivan A., MacLoughlin R. (2021). Evaluation of Aerosol Drug Delivery Options during Adult Mechanical Ventilation in the COVID-19 Era. Pharmaceutics.

[30] Dugernier J., Reychler G., Wittebole X., Roeseler J., Depoortere V., Sottiaux T., Michotte J.B., Vanbever R., Dugernier T., Goffette P. (2016). Aerosol delivery with two ventilation modes during mechanical ventilation: A randomized study. Ann. Intensive Care.

[31] Guillon A., Mercier E., Lanotte P., Haguenoer E., Darrouzain F., Barc C., Sarradin P., Si-Tahar M., Heuzé-Vourc’h N., Diot P. (2015). Aerosol Route to Administer Teicoplanin in Mechanical Ventilation: In Vitro Study, Lung Deposition and Pharmacokinetic Analyses in Pigs. J. Aerosol Med. Pulm. Drug Deliv..

[32] Dhand R. (2003). Aerosol therapy during mechanical ventilation: Getting ready for prime time. Am. J. Respir. Crit. Care Med..

[33] Ari A., Harwood R., Sheard M., Alquaimi M.M., Alhamad B., Fink J.B. (2016). Quantifying Aerosol Delivery in Simulated Spontaneously Breathing Patients with Tracheostomy Using Different Humidification Systems with or Without Exhaled Humidity. Respir. Care.

[34] Montigaud Y., Georges Q., Leclerc L., Clotagatide A., Louf-Durier A., Pourchez J., Prevot N., Perinel-Ragey S. (2023). Impact of gas humidification and nebulizer position under invasive ventilation: Preclinical comparative study of regional aerosol deposition. Sci. Rep..

[35] Ari A., Areabi H., Fink J.B. (2010). Evaluation of aerosol generator devices at 3 locations in humidified and non-humidified circuits during adult mechanical ventilation. Respir. Care.

[36] Hou H., Xu D., Dai B., Zhao H., Wang W., Kang J., Tan W. (2022). Position of different nebulizer types for aerosol delivery in an adult model of mechanical ventilation. Front. Med..

[37] Lee Y.H., Kwon G.Y., Park D.Y., Bang J.Y., Jang D.M., Lee S.H., Lee E.K., Choi B.M., Noh G.J. (2016). Efficiency of a New Mesh-Type Nebulizer (NE-SM1 NEPLUS) for Intrapulmonary Delivery of Ipratropium Bromide in Surgical Patients. Basic Clin. Pharmacol. Toxicol..

[38] Ashraf S., McPeck M., Cuccia A.D., Smaldone G.C. (2020). Comparison of Vibrating Mesh, Jet, and Breath-Enhanced Nebulizers During Mechanical Ventilation. Respir. Care.

[39] Feng Z., Han Z., Wang Y., Guo H., Liu J. (2024). Comparison of the Application of Vibrating Mesh Nebulizer and Jet Nebulizer in Chronic Obstructive Pulmonary Disease: A Systematic Review and Meta-analysis. Int. J. Chronic Obstr. Pulm. Dis..

[40] Ari A., Atalay O.T., Harwood R., Sheard M.M., Aljamhan E.A., Fink J.B. (2010). Influence of nebulizer type, position, and bias flow on aerosol drug delivery in simulated pediatric and adult lung models during mechanical ventilation. Respir. Care.

[41] Zhou Y., Ahuja A., Irvin C.M., Kracko D., McDonald J.D., Cheng Y.S. (2005). Evaluation of nebulizer performance under various humidity conditions. J. Aerosol Med..

[42] Harvey C.J., O’Doherty M.J., Page C.J., Thomas S.H., Nunan T.O., Treacher D.F. (1995). Effect of a spacer on pulmonary aerosol deposition from a jet nebuliser during mechanical ventilation. Thorax.

[43] Thomas S.H., O’Doherty M.J., Fidler H.M., Page C.J., Treacher D.F., Nunan T.O. (1993). Pulmonary deposition of a nebulised aerosol during mechanical ventilation. Thorax.

[44] Mittal G., Kumar N., Rawat H., Chopra M.K., Bhatnagar A. (2010). A radiometric study of factors affecting drug output of jet nebulizers. Indian J. Pharm. Sci..

[45] Liu C.Y., Ko H.K., Fink J.B., Wan G.H., Huang C.C., Chen Y.C., Lin H.L. (2019). Size Distribution of Colistin Delivery by Different Type Nebulizers and Concentrations During Mechanical Ventilation. Pharmaceutics.

[46] Loffert D.T., Ikle D., Nelson H.S. (1994). A comparison of commercial jet nebulizers. Chest.

