# Supplementary material for: In Vitro and In Silico Evaluation of Polymyxin B Aerosol Delivery in Adult Mechanical Ventilation
Source: Pharmaceutics. 2025 Dec 31;18(1):58. doi: 10.3390/pharmaceutics18010058 (PMC12845456; doi:10.3390/pharmaceutics18010058)
Supplement: Supplementary file 1 [file pharmaceutics-18-00058-s001.zip › pharmaceutics-4039796-supplementary.pdf]

Supplementary Material

S1. Materials, Reagents, and Experimental Apparatus

Detailed information on the materials, reagents, and experimental apparatus used in this study is summarized below for enhanced clarity.

Table S1. Materials and reagents used in the study.

Material/Reagent	Manufacturer	Specifications/Description
Polymyxin B sulfate for injection	Shanghai No. 1 Biochemical & Pharmaceutical Co., Ltd., Shanghai, China	500,000 IU/vial
Sterile 0.9% sodium chloride injection	Hunan Kelun Pharmaceutical Co., Ltd., Hunan, China	500 mL bag
Ultrapure water	Merck, Darmstadt, Germany	Ultrapure grade
HPLC-grade methanol	Merck, Darmstadt, Germany	HPLC grade
HPLC-grade acetonitrile	Merck, Darmstadt, Germany	HPLC grade
Endotracheal tube (ETT)	Well Lead Medical Co., Ltd., Guangzhou, China	Adult model, 28 cm length, 7 mm internal diameter
Tracheostomy tube (TT)	Wel lead Medical Equipment Co., Ltd., Guangzhou, China	Adult model, 10 cm length, 7 mm internal diameter
Collection filter	Beijing Huironghe Technology Co., Ltd., Beijing, China	65 mm polypropylene fiber filter (Model: HRHNXM)

Table S2. Experimental apparatus and their purposes in the study.

Device/Instrument	Manufacturer	Purpose in the Experimental Setup
Mechanical ventilator with dual-limb circuit and heated humidifier	Dräger Evita 4 (Dräger, Lübeck, Germany); G-316002 (VADI, Taiwan, China); VHB10 (Inspired Medical, Hongkong, China)	To simulate clinical invasive mechanical ventilation conditions
Breathing simulator	HRH-BRM2100 (Beijing Huironghe Technology Co., Ltd., Beijing, China)	To reproduce spontaneous breathing waveforms and respiratory patterns of critically ill patients
Next Generation Impactor (NGI)	HRH-ZJQ160 (Beijing Huironghe Technology Co., Ltd., Beijing, China)	To determine aerodynamic particle size distribution parameters (MMAD, GSD, FPF, and FPD)
Laser diffraction system	HELOS/INHALER module (Sympatec GmbH, Clausthal-Zellerfeld, Germany)	To measure real-time volume-based particle size distribution (X10, X50, X90, and volume ≤ 5 μm)
High-performance liquid chromatography (HPLC) system	Shimadzu LC-2050 with C18 column (150 × 4.6 mm, 5 μm) (Shimadzu, Kyoto, Japan)	To quantify polymyxin B concentrations in collected samples
Vibrating mesh nebulizer	GUN-300vt (GENTEC, Shanghai, China)	To generate aerosol for delivery evaluation
Jet nebulizer	TL-8 (Bairui Medical Devices Co., Ltd., Changzhou, China) with PARI Junior BOY compressor	To generate aerosol for delivery evaluation (compressor provided 5.1 L/min flow)

S2. Data Extraction and Processing

Characteristic respiratory parameters data were sourced from the MIMIC-IV database (Medical Information Mart for Intensive Care IV), jointly developed by Massachusetts Institute of Technology and Beth Israel Deaconess Medical Center. This de-identified database contains approximately 95,000 ICU patient records from 2008 onward, including vital signs, laboratory tests, medication records, mechanical ventilation parameters, and clinical outcome.

Using Structured Query Language, an initial cohort of 26,568 patients diagnosed with pneumonia was retrieved. Sequential exclusion criteria were applied (see Figure S1): (1) 123 patients with non-infectious pneumonia (e.g., rheumatic pneumonia, idiopathic interstitial pneumonia) were excluded; (2) 13,229 patients who were not admitted to the ICU or had incomplete medical records were excluded. Ultimately, 13,216 ICU patients with confirmed pulmonary infection were included for analysis. The screening process is illustrated in Figure S1.

Considering the multiple repeated measurements typical of ICU patients, the median value was used to represent each patient's parameter level across time points. Data points falling outside mean ± 3 standard deviations were flagged as potential outliers. Obvious measurement or entry errors were removed after clinical review to ensure data quality.

S3. Respiratory Parameters of Critically Ill Patients with Pulmonary Infection

Mechanical ventilation parameters significantly influence aerosol drug distribution and deposition in the lungs of ICU patients. Based on the MIMIC-IV database, this study found considerable inter-individual variability in respiratory patterns among critically ill patients with pulmonary infection. Compared with healthy individuals, patients exhibited decreased tidal volumes (mean: 448 mL) and increased respiratory rates (mean: 21 breaths/min), likely compensatory responses to hypoxemia or hypercapnia. The inspiratory-to-expiratory (I:E) ratio averaged approximately 1:3.2, reflecting prolonged

expiratory phases which may improve gas exchange or reduce gas trapping. These alterations may directly affect aerosol delivery: elevated respiratory rates increase aerosol collision within ventilator circuits, and the lower I:E ratio (longer expiration) may shorten aerosol residence time and increase expiratory losses, thereby reducing effective pulmonary drug delivery.

Table S3 summarizes the key ventilatory parameters of the study population derived from MIMIC-IV data. Given these characteristic respiratory patterns, corresponding parameters were used to configure the breathing simulator for in vitro experiments to assess aerosol pulmonary delivery under clinically relevant conditions.

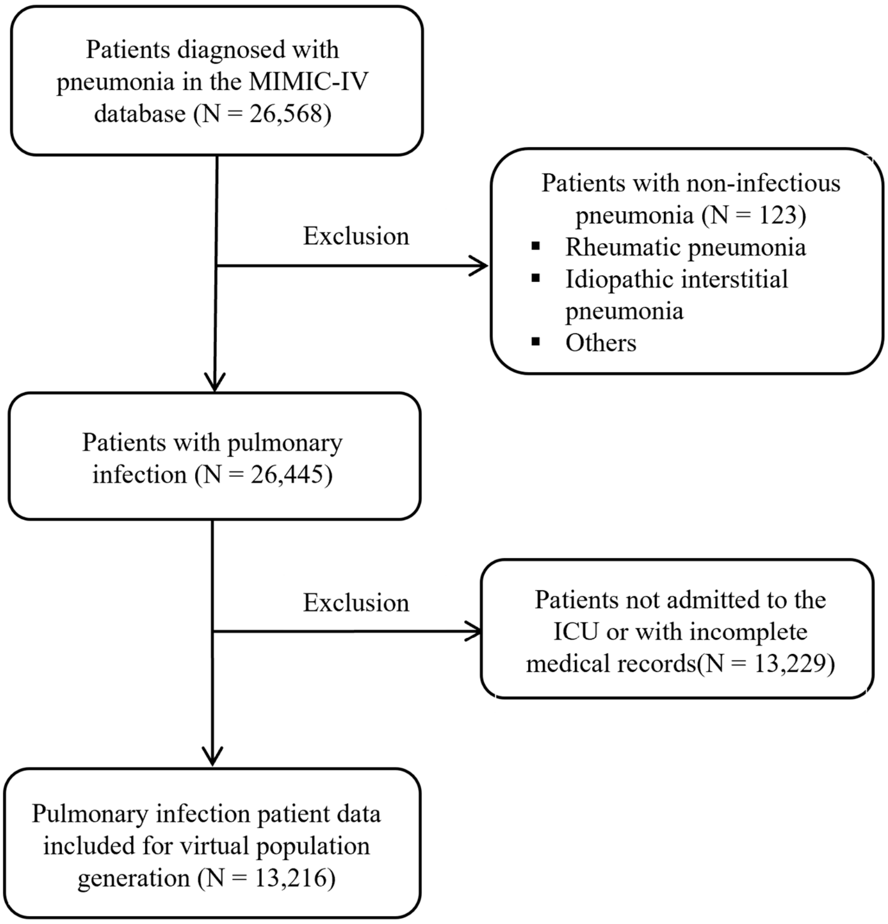

Figure S1. Flowchart of patient extraction from MIMIC-IV database.

Table S3. Respiratory parameters of critically ill patients with pulmonary infection from MIMIC-IV database.

Parameter	Mean	SD	Median
Respiratory rate (breaths/min)	21.09	4.71	20.00
Tidal volume (mL)	448.14	86.56	447.50
Inspiratory:Expiratory ratio	0.31	0.006	0.31
Minute ventilation (L/min)	9.06	2.25	8.80
Positive end-expiratory pressure (cmH_2O)	6.70	2.50	5.00
Plateau pressure (cmH_2O)	19.55	4.50	19.00
Flow rate (L/min)	44.93	9.98	43.80

Abbreviations: SD, Standard deviation

S4. Statistical Analysis of Delivered Dose under Mechanical Ventilation

A three-way Analysis of Variance (ANOVA) was conducted to systematically investigate the influence of nebulizer type, its placement position, and the type of artificial airway on the delivered dose of the aerosolized drug during mechanical ventilation. As summarized in the main text (Section 3.1), the analysis revealed significant main effects for all three factors. Notably, all two-way interactions and the three-way interaction were also found to be statistically significant. This indicates a complex, multifactorial relationship where the effect of any single factor on drug delivery is dependent on the specific combination of the other two factors. Table S4 provides the complete statistical output from this analysis, including F-statistics, p-values, and generalized eta squared as a measure of effect size, thereby substantiating the complex interdependencies observed.

Table S4. Results of three-way ANOVA for delivered dose under mechanical ventilation.

**Effect**	**DFn**	**DFd**	**F**	***p***	**ges**
Nebulizer	1	16	202.312	$1.69*10^{-10}$	0.927
Position	1	16	129.007	$4.54*10^{-09}$	0.89
Airway	1	16	33.472	$2.79*10^{-05}$	0.677
Nebulizer:Position	1	16	20.683	$3.29*10^{-04}$	0.564
Nebulizer:Airway	1	16	7.569	$1.40*10^{-02}$	0.321
Position:Airway	1	16	21.854	$2.54*10^{-04}$	0.577
Nebulizer:Position:Airway	1	16	8.823	$9.00*10^{-03}$	0.355

Abbreviations: DFn, numerator degrees of freedom; DFd, denominator degrees of freedom; F, F-statistic; *p*, *p*-value; ges, generalized eta squared (measure of effect size).

S5. Statistical Analysis of Aerodynamic Particle Size Distribution (APSD)

To assess the impact of nebulizer type and its placement position on the aerosol's physical characteristics, a series of two-way ANOVAs were performed on key aerodynamic parameters measured by the next generation impactor (NGI). The results, referenced in Section 3.4 of the main text, showed that the main effects of both nebulizer type and position, as well as their interaction, were statistically significant across all measured parameters: Mass Median Aerodynamic Diameter (MMAD), Geometric Standard Deviation (GSD), Fine Particle Fraction (FPF), Fine Particle Dose (FPD), and the total dose delivered to the impactor. Table S5 presents the detailed statistical outputs for each of these analyses. The consistent significance of the interaction term (Nebulizer:Position) across all parameters underscores the necessity of the simple effects analysis to properly interpret these results and understand how the performance profile of each nebulizer is altered across different positions.

Table S5. Results of two-way ANOVA for aerodynamic particle size distribution parameters.

Parameter	Effect	DFn	DFd	F	*p*	ges
MMAD	Nebulizer	1	12	40.199	$3.72*10^{-05}$	0.77
	Position	2	12	596.855	$9.72*10^{-13}$	0.99
	Nebulizer:Position	2	12	48.66	$1.75*10^{-06}$	0.89
GSD	Nebulizer	1	12	48.697	$1.48*10^{-05}$	0.802
	Position	2	12	400.059	$1.04*10^{-11}$	0.985
	Nebulizer:Position	2	12	5.756	$1.80*10^{-02}$	0.49
FPF	Nebulizer	1	12	81.921	$1.04*10^{-06}$	0.872
	Position	2	12	530.157	$1.96*10^{-12}$	0.989
	Nebulizer:Position	2	12	68.71	$2.68*10^{-07}$	0.92
FPD	Nebulizer	1	12	28.349	$1.81*10^{-04}$	0.703
	Position	2	12	336.472	$2.89*10^{-11}$	0.982
	Nebulizer:Position	2	12	42.497	$3.59*10^{-06}$	0.876
Delivered dose	Nebulizer	1	12	43.15	$2.66*10^{-05}$	0.782
	Position	2	12	654.425	$5.62*10^{-13}$	0.991
	Nebulizer:Position	2	12	26.032	$4.32*10^{-05}$	0.813

Abbreviations: MMAD, Mass Median Aerodynamic Diameter; GSD, Geometric Standard Deviation; FPF, Fine Particle Fraction; FPD, Fine Particle Dose; DFn, numerator degrees of freedom; DFd, denominator degrees of freedom; F, F-statistic; *p*, *p*-value; ges, generalized eta squared (measure of effect size).

S6. Statistical Analysis of Median Particle Size (X50) Determined by Laser Diffraction

A three-way analysis of variance (ANOVA) was conducted to examine the effects of nebulizer type, placement position, and artificial airway type on the median particle size (X50) measured by laser diffraction during mechanical ventilation. Significant main effects were observed for nebulizer type and artificial airway type, with placement position approaching statistical significance ($p = 0.079$). Notably, the analysis revealed significant two-way interactions, dominated by a particularly strong Nebulizer × Position interaction, indicating that the influence of nebulizer type on X50 was highly dependent on placement position. The three-way interaction was not significant, suggesting that the two-way dependencies were consistent across airway types. These results highlight the

complex interplay among factors in determining in-line aerosol particle size. Full statistical details are provided in Table S6.

Table S6. Three-way ANOVA results for median particle size (X50) from laser diffraction analysis.

Effect	DFn	DFd	F	*p*	ges
Nebulizer	1	64	206.08	$1.11*10^{-21}$	0.763
Position	1	64	3.183	$7.90*10^{-02}$	0.047
Airway	1	64	57.046	$1.98*10^{-10}$	0.471
Nebulizer:Position	1	64	386.418	$8.15*10^{-29}$	0.858
Nebulizer:Airway	1	64	11.009	$1.00*10^{-03}$	0.147
Position:Airway	1	64	15.879	$1.76*10^{-04}$	0.199
Nebulizer:Position:Airway	1	64	1.175	$2.83*10^{-01}$	0.018

Abbreviations: DFn, numerator degrees of freedom; DFd, denominator degrees of freedom; F, F-statistic; *p*, *p*-value; ges, generalized eta squared (measure of effect size).

Owing to the significant Nebulizer × Position interaction identified in the three-way ANOVA, simple effects analyses were performed to clarify pairwise differences within the mechanical ventilation conditions. Additionally, to evaluate the impact of circuit integration relative to optimal standalone performance, each in-line configuration was compared against its respective nebulizer-specific standalone baseline using Dunnett's multiple comparison test. A direct comparison between the two nebulizers under standalone conditions was conducted via independent t-test. Detailed results are presented in Table S7.

Table S7. Simple effects analyses and baseline comparisons for median particle size (X50) from laser diffraction.

Comparison and contrast	Mean difference (SE)	Test statistic (df)	*p*-value
Part A. Pairwise comparisons within mechanical ventilation (from three-way ANOVA)			
1. Nebulizer effect (JN vs. VMN)			
At Y-piece (15 cm) (averaged over airway)	0.567 (0.024)	t (64) = 24.05	<0.0001
At humidifier dry end (averaged over airway)	−0.088 (0.024)	t (64) = −3.75	0.0004
2. Position effect (Humidifier dry end vs. Y-piece 15 cm)			
For ETT (averaged over nebulizer)	0.037 (0.024)	t (64) = 1.56	0.1246
For TT (averaged over nebulizer)	−0.096 (0.024)	t (64) = −4.08	0.0001
3. Airway effect (ETT vs. TT)			
For JN (averaged over position)	0.294 (0.024)	t (64) = 12.50	<0.0001
For VMN (averaged over position)	0.184 (0.024)	t (64) = 7.81	<0.0001
Part B. Mechanical ventilation vs. nebulizer baseline (Dunnett's test)			
1. VMN nebulizer			
Y-piece (15 cm) + ETT vs. standalone	−0.351 (0.027)	t (40) = −13.04	<0.0001
Humidifier dry end + ETT vs. standalone	−0.042 (0.027)	t (40) = −1.57	0.3461
Y-piece (15 cm) + TT vs. standalone	−0.470 (0.027)	t (40) = −17.46	<0.0001
Humidifier dry end + TT vs. standalone	−0.064 (0.027)	t (40) = −2.39	0.0725
2. JN nebulizer			
Y-piece (15 cm) + ETT vs. standalone	−1.07 (0.041)	t (40) = −26.44	<0.0001
Humidifier dry end + ETT vs. standalone	−1.46 (0.041)	t (40) = −35.86	<0.0001
Y-piece (15 cm) + TT vs. standalone	−1.34 (0.041)	t (40) = −32.98	<0.0001
Humidifier dry end + TT vs. standalone	−1.55 (0.041)	t (40) = −38.24	<0.0001
Part C. Direct comparison under standalone nebulizer (independent t-test)			
JN vs. VMN	—	t (9.70) = 35.30	<0.0001

Abbreviations: SE, standard error; JN, jet nebulizer; VMN, vibrating mesh nebulizer; ETT, endotracheal tube; TT, tracheostomy tube.

S7. Statistical Analysis of Proportion of Particles ≤5 μm Determined by Laser Diffraction

A parallel three-way ANOVA was performed to assess the effects of nebulizer type, placement position, and artificial airway type on the volume proportion of particles ≤5 μm (a key indicator of respirable fraction). Significant main effects emerged for all three factors, with nebulizer type exhibiting the largest effect size. Significant two-way interactions were observed, again dominated by a strong Nebulizer × Position interaction, together with notable Position × Airway and Nebulizer × Airway interactions. The three-way interaction was not significant. These findings underscore substantial modulation of the respirable fraction by factor combinations, particularly the marked dependence of nebulizer performance on placement position. Complete results are shown in Table S8.

Table S8. Statistical analysis of proportion of particles ≤5 μm determined by laser diffraction.

**Effect**	**DFn**	**DFd**	**F**	***p***	**ges**
Nebulizer	1	64	424.327	$6.10*10^{-30}$	0.869
Position	1	64	11.865	$1.00*10^{-03}$	0.156
Airway	1	64	124.227	$1.24*10^{-16}$	0.660
Nebulizer:Position	1	64	331.537	$5.27*10^{-27}$	0.838
Nebulizer:Airway	1	64	6.103	$1.60*10^{-02}$	0.087
Position:Airway	1	64	29.655	$8.76*10^{-07}$	0.317
Nebulizer:Position:Airway	1	64	0.009	$9.23*10^{-01}$	$1.47*10^{-04}$

Abbreviations: DFn, numerator degrees of freedom; DFd, denominator degrees of freedom; F, F-statistic; *p*, *p*-value; ges, generalized eta squared (measure of effect size).

Following the significant Nebulizer × Position interaction, simple effects analyses were conducted to delineate specific pairwise differences under mechanical ventilation. In-line configurations were also compared against their respective standalone baselines using Dunnett's test, with a direct standalone nebulizer comparison performed via independent t-test. Detailed outcomes are presented in Table S9.

Table S9. Simple effects analysis and baseline comparisons for proportion of particles ≤5 μm determined by laser diffraction.

Comparison and contrast	Mean difference (SE)	Test statistic (df)	*p*-value
Part A. Pairwise comparisons within mechanical ventilation (from three-way ANOVA)			
1. Nebulizer effect (JN vs. VMN)			
At Y-piece (15 cm) (averaged over airway)	-13.435 (0.490)	t (64) = -27.44	<0.0001
At humidifier dry end (averaged over airway)	-0.828 (0.490)	t (64) = -1.69	0.0958
2. Position effect (Humidifier dry end vs. Y-piece 15 cm)			
For ETT (averaged over nebulizer)	3.078 (0.490)	t (64) = 6.29	<0.0001
For TT (averaged over nebulizer)	-0.693 (0.490)	t (64) = -1.41	0.1619
3. Airway effect (ETT vs. TT)			
For ETT (averaged over position)	-7.987 (0.490)	t (64) = -16.31	<0.0001
For TT (averaged over position)	-6.276 (0.490)	t (64) = -12.82	<0.0001
Part B. Mechanical ventilation vs. nebulizer baseline (Dunnett's test)			
1. VMN nebulizer			
Y-piece (15 cm) + ETT vs. standalone	11.98 (0.701)	t (40) = 17.08	<0.0001
Humidifier dry end + ETT vs. standalone	8.79 (0.701)	t (40) = 12.53	<0.0001
Y-piece (15 cm) + TT vs. standalone	16.90 (0.701)	t (40) = 24.10	<0.0001
Humidifier dry end + TT vs. standalone	9.87 (0.701)	t (40) = 14.08	<0.0001
2. JN nebulizer			
Y-piece (15 cm) + ETT vs. standalone	16.80 (0.606)	t (40) = 27.79	<0.0001
Humidifier dry end + ETT vs. standalone	26.20 (0.606)	t (40) = 43.22	<0.0001
Y-piece (15 cm) + TT vs. standalone	23.40 (0.606)	t (40) = 38.63	<0.0001
Humidifier dry end + TT vs. standalone	29.10 (0.606)	t (40) = 47.94	<0.0001
Part C. Direct comparison under standalone nebulizer (independent t-test)			
JN vs. VMN	—	t (11.8) = -39.9	<0.0001

Abbreviations: SE, standard error; JN, jet nebulizer; VMN, vibrating mesh nebulizer; ETT, endotracheal tube; TT, tracheostomy tube.
